# An Atypical Presentation with Diagnostic Challenge of a Large Cell Neuroendocrine Cancer of Lung: A Case Report and Review of the Literature

**DOI:** 10.4061/2011/912098

**Published:** 2011-06-14

**Authors:** Pavan Kumar Bhamidipati, Amanda Ribbeck, Goldees Liaghati-Nasseri, Ramesh Kumar, Babu Paidipaty B, John Bartnik

**Affiliations:** ^1^Internal Medicine, Synergy Medical Education Alliance, 1000 Houghton Avenue, Saginaw, MI 48602, USA; ^2^Pulmonary and Critical Care, Synergy Medical Education Alliance, 1000 Houghton Avenue, Saginaw, MI 48602, USA; ^3^Hematology and Oncology, Oncology Hematology Associates of Saginaw Valley, 5400 Mackinaw Road, Suite 4200, Saginaw, MI 48604-9533, USA

## Abstract

Large-cell neuroendocrine carcinomas (LCNECs) are relatively rare and aggressive neoplasms of the lung with very poor prognosis. Even though they are included in the classification of nonsmall cell carcinomas, they have a biological behaviour and physiological response to treatment more like small cell carcinomas of lung. We report an atypical case presentation of LCNEC in a 51-year-old gentleman who presented with diffuse metastases to the thoracic and lumbar spine, brain, and liver, posing a diagnostic challenge. The primary small central lung tumor was in close proximity to major vessels, rendering a biopsy of the primary cancer challenging and nearly impossible. The final diagnosis was established through immunohistochemistry staining and examination of liver biopsy from a metastatic lesion. We also included a review of the current literature pertinent to LCNEC, as well as the important role of tumor markers plus immunohistochemistry profiles in determining the origin of unknown primary tumors in such difficult patient presentations.

## 1. Introduction

Large cell neuroendocrine carcinoma is a rare and aggressive neoplasm of lung with a very poor prognosis. It accounts for approximately 1.6–3.1% of all lung cancers [1]. Most LCNECs present as large primary lung masses in the peripheral lung fields; they are more frequently identified on chest radiographs [2]. Patients with LCNEC are less likely to present with pulmonary symptoms such as cough, hemoptysis, or postobstructive pneumonia [3]. Overall, prognosis for the present patient after diagnosis of stage IV LCNEC with distant metastasis was poor, and life expectancy was estimated at around six months. Diagnosis of LCNEC is often a difficult task, which requires histological analysis, cytological evaluation as well as immunohistochemistry. To confirm neuroendocrine origin in the tumor cells, at least one immunohistochemical marker, such as chromogranin, synaptophysin, or CD56, must be positive [4]. Based on the biological presentation and behavior, LCNEC actually has similar prognosis and is treated with similar management regimes as small cell carcinoma [5–7]. 

We are going to present a case of atypical presentation of LCNEC where the tumor presented with diffuse and distant metastases to liver, spine, brain and including adrenal gland that posed a diagnostic challenge in a 51-year-old gentleman. We also included a small review of the tumor markers that aided in the diagnosis of this tumor.

## 2. Case Presentation

A 51-year-old Caucasian male presented to the emergency department with a two-week history of lower back pain and an episode of bowel and bladder incontinence and increasing confusion. He tried analgesics with no symptomatic relief. He also experienced a 20-pound weight loss in three weeks. Pertinent social history includes a 34-pack year history of tobacco smoking, as well as history of heavy alcohol consumption in the past. He denied any pulmonary symptoms at presentation. 

**Figure 1:**
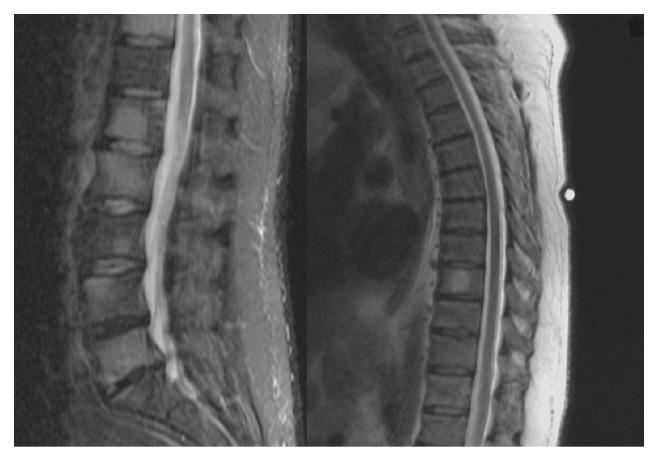
MRI T2 images showing thoracic and lumbar vertebral metastatic disease.

**Figure 2:**
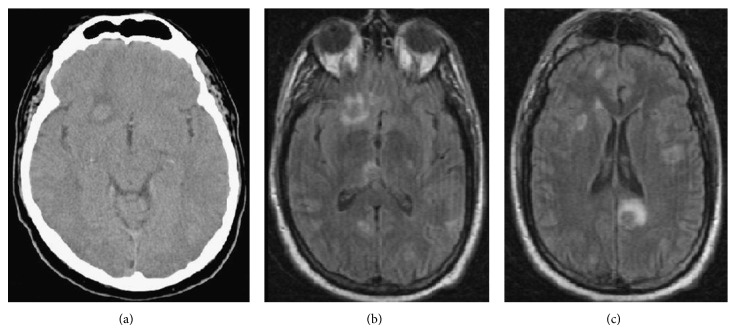
(a) Ct Brain 1.5 × 1.16 cm hemorrhagic right frontal lobe metastatic disease. (b, c) Postgadolinium MRI shows multiple ring enhancing metastatic lesions.

MRI of lumbar and thoracic spine at presentation showed diffuse marrow replacement by hyperdense lesions at multiple vertebral levels and surrounding edema, with complete spinal cord sparing. This finding was highly suggestive of neoplastic metastases (Figure 1). Subsequent brain CT and MRI showed multiple edematous hyperdense lesions, also consistent with metastatic disease (Figures 2(a), 2(b), and 2(c)). 

**Figure 3:**
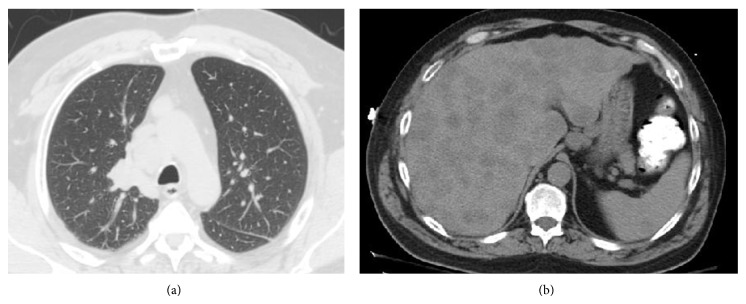
(a) CT of chest with contrast in mediastinal view displaying 2 cm right suprahilar soft tissue mass. (b) CT of abdomen showing multiple hepatic metastatic foci.

Chest X-ray was then performed, revealing prominence and opacity of the soft tissue near the right paratracheal region. Upon closer examination, CT showed a 2 cm right suprahilar soft tissue mass suggestive of a central pulmonary neoplasm with surrounding atelectasis, marked mediastinal and right hilar lymphadenopathy, numerous low-density hepatic lesions suspicious of metastatic disease, abdominal lymphadenopathy, and a left adrenal nodule suggestive of metastatic disease (Figures 3(a) and 3(b)). There were no other gastrointestinal lesions noted.

**Figure 4:**
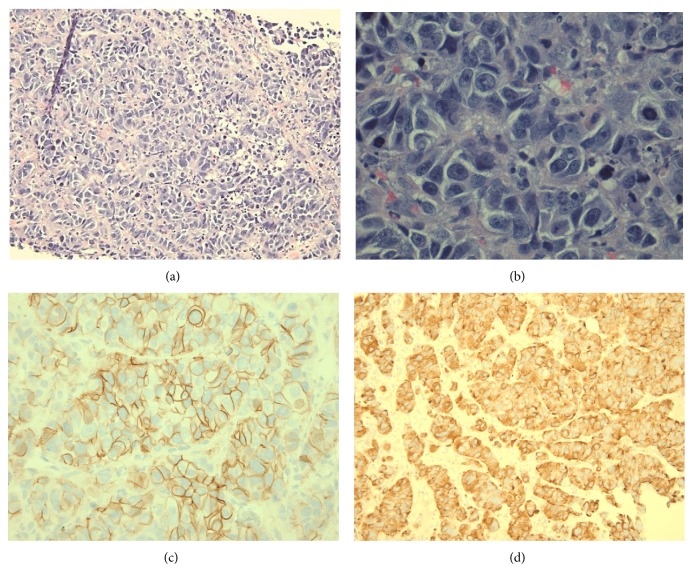
(a) Liver Biopsy—light microscopy at 200x magnification of metastatic lesion. (b) Liver Biopsy—light microscopy at 600x magnification of metastatic lesion. (c) Liver Biopsy—positive immunostaining with CD56. (d) Liver Biopsy—positive immunostaining with chromogranin.

Biopsy of a metastatic liver lesion was chosen for histological examination due to its accessibility and ease of aspiration. Cytological examination showed diffuse sheets of large, aggressive cells with multiple mitotic figures per high power field. The cuboidal shaped cells also consistently demonstrated increased nuclear/cytoplasmic ratios with numerous prominent nucleoli (Figures 4(a) and 4(b)). Subsequent immunohistochemistry revealed cells staining positive for cytokeratin-7, chromogranin, and CD56, but negative for cytokeratin-20 and Thyroid Transcription Factor 1 (TTF-1) (Figures 4(c) and 4(d)). Final pathology report was consistent with high-grade large cell neuroendocrine carcinoma. 

He was categorized as extensive-stage (Stage IV) LCNCE. The patient currently undergoing focal radiation to the spine and brain, augmented with chemotherapy regimen of Paclitaxel and Carboplatin every 28 days. In view of uncertainties in the responsiveness of these tumors for various chemotherapeutic agents, the above agents were chosen. In view of his centrally located tumor surrounded by major vessels, biopsy was not an option for this patient. Unfortunately, due to advanced nature of his disease and aggressiveness of this tumor, his prognosis was considered poor.

## 3. Discussion

The World Health Organization first elucidated large cell neuroendocrine carcinoma as its own entity in the late 1990s [2], and the classifications of pulmonary neuroendocrine tumors have changed dramatically since first encountered. Large cell neuroendocrine carcinoma (LCNEC) accounts for approximately 1.6–3.1% of all lung cancers [1]. 

The present case demonstrates a patient who presented with diffuse metastasis and a small, central primary lung lesion without any pulmonary symptoms. Upon radiological examination, the patient was determined to have stage IV metastatic disease. Patients with LCNEC are less likely to present with pulmonary symptoms such as cough, hemoptysis, or postobstructive pneumonia [3]. Zacharias et al. reported that only 4 out of 21 patients presented with cough or hemoptysis, while the remainder of patients presented with asymptomatic nodules, chest pain, or nonspecific flu-like symptoms [8]. The unusual centrally located primary LCNEC lung lesion in this patient was found in close proximity to the pulmonary vessels, which could have facilitated in his rapid and distant spread of metastases without the presence of an evidently large primary tumor. This could have been a reason for the patient's late presentation and subsequent rapid decline. A centrally located primary pulmonary lesion is a relatively rare finding in LCNEC, as Garcia-Yuste et al. reported that two thirds of LCNECs present in the periphery of the lung parenchyma [9]. In a subsequent case series by Paci et al., only 1 out of 48 LCNEC was found to originate from a central location [10]. 

Diagnosis of LCNEC is often a difficult task, which requires histological analysis, cytological evaluation, as well as immunohistochemistry. Since most LCNEC present as large primary lung masses in the peripheral lung fields, they are more frequently identified on chest radiographs [2]. Because of their peripheral location, diagnosis is most often made by transthoracic fine needle aspiration biopsy due to its ease of accessibility [2]. In this case, however, the patient presented with a small centrally located primary mass in close proximity to the pulmonary vessels, making transthoracic fine aspiration or bronchoscopy difficult and hazardous. Since the patient had wide and distant metastasis with extensive hepatic involvement, the biopsy route via hepatic fine needle aspiration was chosen due to the ease of procedure and minimal discomfort accorded to our patient. 

Morphologically, LCNEC tumors cells often show features of basaloid palisading, trabecular growth patterns, rosette formation, and organoid nesting [11]. The malignant cells are large with moderate to abundant eosinophilic cytoplasm, high N/C ratios, and numerous prominent nucleoli, easily distinguishing the cells from those of small cell carcinoma upon first inspection [1]. Mitotic counts typically exceed 10 per 10 HPF of viable tumor [7]. All of these features are evidenced in the following slides.

To confirm neuroendocrine origin in the tumor cells, at least one immunohistochemical marker, such as chromogranin, synaptophysin, or CD56, must be positive [9]. The current patient presented with both CD56 and chromogranin positivity, confirming neuroendocrine origin. CD56, also known as neural cell adhesion molecule (NCAM), is a cell-surface protein involved in cell-to-cell interactions during neural development and is the most sensitive and specific marker in confirming neuroendocrine differentiation in malignant neoplasms [12]. Chromogranin A (CgA), a secretory protein involved in neural cell adhesion and expressed by many neuroendocrine cells, has also been recognized as a useful tissue and serum marker of neuroendocrine tumors [12]. Cytokeratins (CK) 7 and 20 are low molecular weight cytokeratins and their anatomic distribution is generally restricted to epithelia and their neoplasms [13]. CK7 expression is essentially ubiquitous in lung adenocarcinomas (100% of cases), but is also observed in other primary lung carcinomas and is positive in 70% of LCNEC [14]. CK20, on the other hand, shows restricted expression in adenocarcinomas of the gastrointestinal tract and transitional cell carcinomas of the urinary tract, and thus was used in this case to rule out gastrointestinal origin [15]. CK7 positivity in concert with CK20 negativity strongly favors primary lung origin.

Further, Thyroid Transcription Factor-1 (TTF-1) is a nuclear transcription protein in the NKx2 family that regulates development, cell growth, and differentiation in thyroid, lung, and select brain tissue [16, 17]. Positive immunostaining for this protein is present in approximately 40% of all large cell neuroendocrine tumors with origin in the lung [18]. Those with negative staining are often more poorly differentiated [19]. The present case showed TTF-1 negativity, indicating a poorer prognosis and consistent with advanced metastatic disease at presentation. Conclusively, the present immunohistochemical pattern (Chromogranin+, CD56+, CK 7+, TTF-1−, CK 20−), in addition to the aforementioned morphological characteristics, are consistent for the diagnosis of a high-grade large cell neuroendocrine carcinoma. Specifically, CK7 positivity in addition to TTF-1 and CK20 negativity in this patient strongly favors the pulmonary system as the primary site of LCNEC origin; however, as lung biopsy was not performed, we cannot be certain based solely on histological examination. Further, because the histological information was concordant to the patient's clinical presentation and radiology specimens, primary lung LCNEC was reported.

Overall, prognosis for the present patient after diagnosis of stage IV LCNEC with distant metastasis was poor and life expectancy was estimated at around six months. In previous case reports, Travis et al. and Garcia-Yuste et al. reported the 5-year overall survival rate for LCNEC at 27% and 21%, respectively, irrespective of cancer staging [1, 9]. More recent studies from Asamura et al. have shown the 5-year survival rate at 41.3% [20]. This value falls between the range of reported 5-year survival rates of 13–47%, found in multiple recent case reports [10, 21–23]. However, patients with stage IV LCNEC unfortunately have a much more dismal prognosis, with a 5-year reported survival rate of 0% [23]. This low survival rate is likely a result of late patient presentation with multiple distant metastases at the time of diagnosis. 

Based on the biological presentation and behavior, LCNEC actually has similar prognosis and is treated with similar management regimes as small cell carcinoma [5–7]. The response rate of large cell neuroendocrine carcinoma to cisplatin-based chemotherapy was comparable to that of small cell lung carcinoma [24]. The variability in the responsiveness of LCNEC to Etoposide and Taxane group drugs in several small studies makes it difficult to choose the right chemotherapeutic agent for this condition. Even though the survival benefit with Etoposide and Cisplatin is clearly evident with completely resected LCNEC, no clear evidence exists to what chemotherapeutic drugs to use in treatment/palliation for advanced stages. Most patients initially respond to chemotherapeutic agents, but early relapses are frequent and resistant to currently available treatments [24]. However, stage IV patients with poor prognosis, such as the patient presented here, may benefit from more aggressive treatment with such novel chemotherapeutic agents.

## 4. Conclusion

Large cell neuroendocrine carcinoma is a rare and aggressive neoplasm with a very poor prognosis. This case presents an atypical presentation of an asymptomatic patient with vast metastatic disease. In such advanced cases, we must often rely on immunohistochemical markers in order to distinguish a final diagnosis. Chromogranin and NCAM/CD56 are excellent histochemical markers to delineate tumors of neuroendocrine origin. Staining patterns of TTF-1 in combination with multiple cytokeratin marker positivity further allows deduction of primary tumor location and even levels of differentiation of such metastatic tumor cells. 

Our current treatment approaches of chemotherapy and radiotherapy do not significantly improve the outcome in patients diagnosed with stage IV LCNEC presenting with widespread metastasis, rendering novel approaches of management and diagnosis necessary to curb the mortality rates in such rare and aggressive presentations of LCNEC in the lung. 

This rare presentation in the presently described case report elucidates the difficulties in present LCNEC diagnosis, management, associated poor outcomes, and the necessity for more advanced chemotherapeutic options.

##  Consent

Written informed consent was obtained from the patient for publication of this case report and accompanying images. A copy of the written consent is available for review by the Editor-in-Chief of this journal.

##  Conflict of Interests

The authors declare that they have no conflict of interests.

##  Authors' Contributions

G. Liaghati-Nasseri and A. Ribbeck obtained the media for this paper. All the authors reviewed the literature even though GL and AR were the major contributors. All authors read and approved the final manuscript.

## Abbreviations

### Abbreviations

LCNEC: Large cell neuroendocrine carcinoma
SCC: Small cell carcinoma
NSCLC: Nonsmall cell lung cancer
N/C: Nuclear cytoplasmic ratio
CK: Cytokeratin
NCAM: Neural cell adhesion molecule
CgA: Chromogranin A
TTF-1: Thyroid transcription factor-1.
